# Direct and Interactive Effects of Work Stress and Professional Identity on Job Burnout Among Elderly Care Workers for Old People in China: A Cross-Sectional Study

**DOI:** 10.3390/healthcare13141635

**Published:** 2025-07-08

**Authors:** Luqi Li, Qi Wang, Qiaoqiao Wang, Yating Chen, Tao Sun, Caiming Xu, Li Li

**Affiliations:** 1 School of Law, Hangzhou City University, Hangzhou 310015, China; 202211114611002@zcmu.edu.cn (L.L.); wangqi0207@hzcu.edu.cn (Q.W.); xucm@hzcu.edu.cn (C.X.); 2 School of Medicine, Hangzhou City University, Hangzhou 310015, China; wangqq@hzcu.edu.cn; 3 Shcool of Humanities and Management, Zhejiang Chinese Medical University, Hangzhou 310053, China; 20051031@zcmu.edu.cn; 4 School of Public Health, Hangzhou Normal University, Hangzhou 311121, China; hydsuntao@126.com

**Keywords:** work stress, professional identity, job burnout, elderly care workers, interactive effect

## Abstract

**Background**: This study aimed to explore the relationships between work stress, professional identity, and job burnout in elderly care workers and to assess whether work stress and professional identity interact to influence job burnout. **Methods**: A survey of 439 elderly care workers was conducted from July to September 2021. T-tests and ANOVA were used to identify the main different characteristics involved in work stress and professional identity, and four multiple regression models were used to identify the determinants of job burnout. **Results**: Work stress and professional identity were moderate among respondents. Work stress positively correlated with job burnout, while professional identity showed a negative correlation. An interactive effect was found between the sub-dimension of work stress concerning the consistency between rewards and responsibilities and professional identity on job burnout. **Conclusions**: Managers in pension institutions should focus on addressing work stress and professional identity, especially balancing alignment between rewards and responsibilities.

## 1. Introduction

With the growth of population aging, the issue of older care service has attracted widespread attention from the government, society, and scholars. According to the National Bureau of Statistics of China, by the end of 2023, the number of individuals aged 60 and above had reached 312 million, accounting for 21.1% of the total population [1]. As well known, older people often experience a decline in cognitive abilities and physical function, which can threaten their independence. In 2020, an estimated 47 million disabled or semi-disabled older adults lived in China [2]. With the growing need for daily assistance, caregiving has placed significant pressure on both social systems and families. However, shrinking family sizes and the rise of empty-nest households have made traditional family care-giving increasingly inadequate to meet the growing demand for elderly care service. Consequently, the development of a diversified formal elderly care service has become crucial and urgent. The major challenge in this regard is the shortage of elderly caregivers, compounded by difficulties in attracting and retaining qualified professionals [3]. The National Medium- and Long-Term Development Plan for Civil Affairs Personnel (2010–2020) points out that in 2010, there were only 30,000 elderly care workers in China, and this number is expected to increase to 6 million by 2020 [4]. Despite this growth, the high turnover rate among these workers has resulted in a significant workforce gap, estimated to be at least 5 million, due to factors such as low wages, high work stress, and job burnout [5]. In China’s elderly care service, each caregiver takes care of an average of six older people daily. Wu Fang’s study revealed that 24.26% elderly care workers cared for 11 to 20 older people per day. This ratio is notably higher than the common 4:1 ratio in developed countries, which may imply potential challenges in the quality and intensity of care provided [6]. To address this issue, the entry requirements for caregivers have been set relatively lenient, with no formal educational prerequisites, according to the National Occupational Standard for Elderly Caregivers (2019) [7], formulated by China’s Ministry of Labor and Social Security. However, in practice, caregivers are expected to possess a broad range of professional skills, including basic nursing, rehabilitation therapy, mental health support, and emergency response, to provide high-quality care [8]. These extensive job requirements have significantly increased both the workload as well as the physical and emotional stress experienced by elderly care workers [9]. Long-term stress can impact job satisfaction, work quality, and job performance, leading to negative outcomes such as burnout or turnover intentions [10,11]. Therefore, it is important for both elderly care workers and their managers to take measures to reduce work stress.

Work stress has several definitions in a variety of theoretical models [12]. According to the cognitive theory of stress and coping proposed by Lazarus and Folkman, work stress is conceptualized as the dynamic interaction between the individual and their environment [13]. Work stress is also a dynamic relationship between external environmental stimuli and individual responses, resulting from the interaction between the two. It involves both stress sources and individual cognitive evaluations [14]. Generally, the greater the mismatch between external demands and an individual’s capabilities, the more stress they are likely to experience [15,16,17]. Scholars such as Ahlin, et al. have identified that long working hours, overloaded work tasks, and a lack of necessary skills are the primary sources of work stress currently faced by elderly care workers. These factors not only contribute to high levels of burnout and turnover but also compromise the quality of care provided to older people. Additionally, the emotional and physical demands of the job, coupled with inadequate support and training, further exacerbate the challenges faced by this essential workforce [17,18].

In recent years, considerable research has been conducted on the connection between work-related stress and job burnout. Job burnout is characterized as a prolonged response to persistent emotional and interpersonal stressors in the workplace, typically manifested by emotional exhaustion, depersonalization, and reduced personal accomplishment [19,20]. This negative emotional experience not only affects physical and mental health, but also diminishes work efficiency and productivity [21,22]. Xu Jiaming suggests there is obvious job burnout among nursing staff for the age in China and the results of multiple linear regression analysis shows that monthly income level, family attitude, working years and day work time are the influencing factors of job burnout [23]. Other research indicates that the intensifying work pressure and challenges make caregivers more susceptible to burnout [22]. However, it is observed that even under similar stress conditions, the extent of burnout can vary among individuals, indicating that other factors may influence the mitigation of stress effects [24].

According to the Job Demand-Resource model, the demands of a job, such as work load, time pressure, or work interruptions, are connected to the stress and burnout experienced by the employee, whereas the resources of the job, such as received support at the workplace, task autonomy, or professional identity, can reduce stress and can also buffer the effects of job demands on job burnout and turnover intention. Professional identity can be defined as an individual’s recognition and comprehension of the beliefs, values, attitudes, and roles associated with their professional group, which also encompasses one’s self-perception [25,26,27]. It often serves as a mediating or moderating factor. Yang and Fang revealed that professional identity is a critical mediating factor, bridging work-family support to burnout among primary health workers [28]. The partial moderating role of professional identity between the emotional exhaustion dimension of burnout and job satisfaction was found in a survey of Chinese general practitioners [29].

Numerous research has focused on the relationships between job stress, professional identity, and job burnout, which highlighted the significance of work stress and professional identity in alleviating burnout. Professional identity was negatively correlated with job burnout, whereas work stress showed a positive correlation with burnout [30,31,32,33]. However, there was a lack of information on the joint effects of professional identity and work stress on job burnout. Given these consequences, this study aimed to explore the following: (a) the levels of work stress, professional identity, and job burnout that occurs in elderly care workers; (b) the associations among work stress, professional identity, and job burnout; and (c) whether there is an interactive effect of work stress and professional identity on job burnout. The interactive effect refers to the fact that the effects of multiple independent variables on the dependent variable are not simply the sum of their individual effects.

## 2. Materials and Methods

### 2.1. Sample

A cross-sectional survey of elderly care workers was conducted from July to September 2021. A total of 13 cities in Heilongjiang province were selected, and two pension institutions were randomly selected from each city. The research team visited each of the selected pension institutions, in the form of a face-to-face structured interview. Across the 26 sampled institutions, the number of caregivers per facility ranged from 10 to 30. In total, these institutions had 556 eligible participants. To ensure data consistency, caregivers absent on survey days that fell on weekends or holidays were excluded from the sampling process. Questionnaires were administered to all remaining on-duty personnel directly engaged in elderly care work and willing to participate in this study. The questionnaire included a detailed cover page outlining the study purposes, procedures, ethical considerations, and filling instructions. Data were collected anonymously, and respondents completed the questionnaires privately to ensure confidentiality. Respondents were assured that participation was voluntary, and furthermore, the return of questionnaires signified informed consent. A total of 500 questionnaires were distributed, with 87.8% (439 copies) returned as valid responses, reflecting a high effective response rate.

### 2.2. Measures

The survey questionnaire is composed of four sections: basic characteristics, work stress, professional identity, and job burnout. The first two were self-designed scales, while the latter two were international common scales. Section 1 focused on the basic characteristics of the respondents. Meanwhile, based on the two-factor theory, related questions were set to explore factors potentially differentiating work stress and professional identity. Section 2 measured the level of work stress using a self-made evaluation scale with 9 items. Factor analysis (not elaborated in this paper) yielded three subscales that comprised 8 items. The three-subscale solution accounted for 74.42% of the overall variance. These three stress subscales were named “consistency between revenue and responsibility”, “work task”, and “work conditions”. They individually accounted for 37.22%, 21.16%, and 16.02% of the overall variance. For example, “Do you feel stressful by workload or work tasks while caring for older people?” Each item was scored on a scale ranging from 1 (no stress) to 5 (huge stress), and the total score was 40. Higher scores indicated stronger work stress among the respondents. The Kaiser–Meyer–Olkin (KMO) value was 0.83. Section 3 measured professional identity by the professional identity scale developed by Tyler and McCallum [34]. The 10-item scale was based on a 5-point Likert scale, for example, “I strongly agree with my work”. Responses ranged from 1 (completely inconsistent) to 5 (fully compliant), with the total score spanning 10 to 50. Higher scores indicated a stronger professional identity. The scale achieved reasonable reliability in this study (Cronbach’s α = 0.94).

Section 4 assessed elderly care workers’ level of burnout, which was measured by the Maslach-compiled [35] and Chinese-scholar-Li-Chaoping-and-Shi-Kan-revised [36] Ma Burnout Scale (MBI-GS). There are 15 items within the MBI-GS Chinese version, and it is based on Likert7 points. The score ranged from 0 (never happen) to 6 (happens every day). Three subscales emerged from the data analysis: emotional exhaustion (Cronbach’s α = 0.94), depersonalization (Cronbach’s α = 0.94), and personal accomplishment (Cronbach’s α = 0.93). Various psychometric analyzes showed that the scale has both high reliability and validity as a measure of burnout.

### 2.3. Statistical Analysis

In this study, data were analyzed with SPSS (IBM, New York, NY, USA) software, version 25.0. Firstly, descriptive analyzes were performed by frequency (N) and percentage (%) to summarize the demographic characteristics of participants. Secondly, *t*-tests and ANOVA were used to compare the work stress and professional identity of elderly care workers with different characteristics. Thirdly, the means (M) and standard deviations (SD) were calculated to show the scores of work stress, professional identity, and job burnout among the respondents.

Fourth, based on previous research [30], four multiple regression models were employed to determine the factors contributing to job burnout. Each model incorporated the characteristics of the respondents. Model 1 focused on the impact of professional identity. Model 2 evaluated the influence of various subscales of work stress. Model 3 investigated the individual contributions of professional identity and work stress to job burnout. Model 4 explored how the interaction between professional identity and work stress affected job burnout.

## 3. Results

### 3.1. Fundamental Characteristics of Respondents

Table 1 shows the fundamental characteristics of respondents. The majority of them were female (87.7%), married (83.8%), aged between 41 and 60 years (80.2%), junior high school education or below (71.1%), and family members ≤3 people (74%). More than half of the respondents have signed labor contracts with their unit (62.2%), and their income is determined by the number of older people served (58.1%). Monthly earnings were relatively low, with 89.3% making less than 3000 yuan, yet 54.9% expressed satisfaction with their income. The majority had received post-training before taking up the job (80.2%). A considerable worked over 10 h a day (57.9%). Regarding job mobility, 72.4% had never switched pension institutions, and 62.2% self-reported being in good health. When it came to insurance, more than half, 61.5%, had medical insurance, while only 42.4% had pension insurance.

### 3.2. Levels of Work Stress, Professional Identity, and Job Burnout of Elderly Care Workers

Levels of work stress, professional identity, and job burnout were reported in Table 2. Concerning work stress, the consistency between revenue and responsibility (2.49 ± 0.96) subscale of work stress rated the highest, followed by work task (2.08 ± 0.72), and work conditions (1.61 ± 0.62). The average score of professional identity was 3.54 ± 0.74. And in job burnout, the average score of total items was 24.95 ± 12.44. Specifically, the average scores of emotional exhaustion, depersonalization, and personal accomplishment were 8.34 ± 5.06, 5.62 ± 3.78, and 11.00 ± 6.50, with the average item score of 1.67 ± 1.01, 1.40 ± 0.94, and 1.83 ± 1.17, respectively.

### 3.3. Single-Factor Analysis of Work Stress and Professional Identity of Elderly Care Works

In this study, the results of the single-factor analysis are shown in Table 1 as well. Regarding the consistency between the revenue and responsibility subscale of work stress, statistically significant differences were observed in relation to several factors: the number of family members (t = 2.80, *p* < 0.01), income satisfaction (F = 11.96, *p* < 0.01), self-assessment health (F = 5.74, *p* < 0.01) and medical insurance (t = −3.85, *p* < 0.01). The differences of work task subscale of work stress were statistically significant in terms of number of family members (t = 2.51, *p* < 0.05), labor contract signing (t = −3.11, *p* < 0.01), compensation mechanism (F = 3.64, *p* < 0.05), income satisfaction (F = 11.25, *p* < 0.01), number of replace pension institutions (F = 5.11, *p* < 0.01), self-assessment health (F = 9.16, *p* < 0.01) and medical insurance (t = −2.78, *p* < 0.01). When it came to work condition subscale of work stress, there were statistically significant differences associated with work conditions in relation to gender (t = 3.55, *p* < 0.01), number of family members (t = 2.32, *p* < 0.05), monthly income (F = 4.64, *p* < 0.05), compensation mechanism (F = 11.41, *p* < 0.01), income satisfaction (F = 5.15, *p* < 0.01), number of replace pension institutions (F = 4.29, *p* < 0.05), post-training (t = 3.31, *p* < 0.01) and medical insurance(t = −2.29, *p* < 0.05).

There were statistically significant differences in professional identity in terms of age (F = 2.74, *p* < 0.05), number of family members (t = −2.00, *p* < 0.05), compensation mechanism (F = 4.29, *p* < 0.05), income satisfaction (F = 17.25, *p* < 0.01), self-assessment health (F = 31.11, *p* < 0.01), post-training (t = −7.71, *p* < 0.01) and pension insurance (t = 3.71, *p* < 0.01).

### 3.4. Correlation Matrix Between Variables

Table 3 shows the correlation between the three factors of work stress (i.e., consistency between revenue and responsibility, work tasks, and work conditions) and professional identity and job burnout. The results show that there was a strong correlation between the three factors. Work stress was negatively correlated with professional identity and positively correlated with job burnout (*p* < 0.01), while professional identity was negatively correlated with job burnout (*p* < 0.01).

### 3.5. Regression for Overall Job Burnout Among Elderly Care Workers

Table 4 shows four regression models used to assess the key determinants of overall job burnout. In Model 1, inclusion of the professional identity resulted in age and medical insurance emerging as positive predictors of job burnout, while monthly income and professional identity served as negative predictors of overall job burnout (adjusted R^2^ = 0.30). For Model 2, after incorporating the subscales of work stress, age, self-reported health, pension insurance, the “consistency between revenue and responsibility” and “work conditions” subscales of work stress were significant positive predictors of overall job satisfaction. Meanwhile, monthly income and the compensation mechanism were negative predictors of overall job burnout (adjusted R^2^ = 0.39).

In Model 3, with the inclusion of the subscales of work stress and professional identity, the adjusted R^2^ reached 0.45. The results showed that professional identity and the “consistency between revenue and responsibility” and “work conditions” of work stress were statistically significant for job burnout. In model 4, then the scales of professional identity, work stress, and their interactive items were included, and the adjusted R^2^ was 0.46. In this model, we found that the independent effect of work stress on job burnout was no longer significant. In contrast, the professional identity and the interaction of “professional identity*consistency between revenue and responsibility” were significant, with beta values in opposite directions. This indicates that the “consistency between revenue and responsibility” in work stress plays a predominantly interactive role in the influence of professional identity on job burnout. Specifically, under high work stress, professional identity no longer plays a positive predictor of burnout.

## 4. Discussion

### 4.1. Overall Characteristics of Work Stress, Professional Identity, and Job Burnout of Elderly Care Workers

Among elderly care workers, the mean scores for the three dimensions of work stress, in descending order, were consistency between revenue and responsibility, work task, and work conditions. This result shows that the lack of consistency between revenue and responsibility is the prime contributor to work stress. Previous studies showed that open communication between supervisors and employees about stress can prevent this buildup, yet this requires a secure communication environment [37].

The total score for the professional identity of the elderly care workers in this study was 35.38, with an average of 3.54 points, suggesting a relatively high level of professional identity. This finding was similar to Sun, which suggested that the nursing students after a 6-month sub-internship in a general hospital had a positive professional identity [38].

For job burnout among elderly care workers, the mean scores for the three dimensions, from highest to lowest, were personal accomplishment, emotional exhaustion, and depersonalization. This suggests that job burnout is more prominently reflected in personal accomplishment. This was contrasted with Shen’s study, which indicated that the job burnout rate showed 37%, 28%, and 26% for emotional exhaustion, depersonalization, and personal accomplishment, respectively [39]. The finding of Feng’s study was 65.02%, reported for a medium or high level of emotional exhaustion, 35.38% for personal accomplishment, and 62.05% for reduced personal achievement [29]. Consequently, institutions could strengthen the balanced mechanism of revenue and responsibility to alleviate work stress. Additionally, boosting the personal accomplishment of elderly care workers would be beneficial for alleviating job burnout.

### 4.2. Relationships Between Work Stress, Professional Identity, and Job Burnout Among Elderly Care Workers

To explore the factors influencing the changes in job burnout, four distinct multiple regression models were assessed, with specific consideration of work stress and professional identity. The socioeconomic characteristics of elderly care workers were included in all models, and each model accounted for a significant proportion of the variation in job burnout. In Model 1, professional identity, when considered alone, was found to be significant, corroborating previous research [40]. In Model 2, the work stress, when analyzed independently, had a significant greater impact on job burnout compared to Model 1. Tavakoli’s study also confirmed the significant effect of work stress on job burnout [41]. Other studies have similarly verified fragmented, intensive work, and low wages can predict job burnout [42]. Additionally, adverse work conditions, such as job insecurity and confronting with challenging situations, may induce job burnout [43].

Model 3 uses professional identity and work stress together. In this case, age, the compensation mechanism, and professional identity, as well as two aspects of work stress (the work conditions subscale and consistency between revenue and responsibility subscale) emerged as important determinants of job burnout. Lim’s study showed that professional identity moderated the relationship between job stress and burnout among care workers in home care facilities [44].

However, after incorporating the interactive items between work stress and professional identity based on Model 3 (Model 4), professional identity remained a significantly negative predictor of job burnout, while the interactive items “professional identity*consistency between revenue and responsibility” was significantly positive in predicting job burnout. This indicates that work stress moderates the impact of professional identity on job burnout. Hence, more attention should be paid to the work stress of elderly care workers to alleviate the occurrence of job burnout. Furthermore, Maor’s study indicated that there was a significant interaction between professional identity and work stress regarding job burnout [30]. Heo’s study confirmed that the correlation between work stress and job burnout is stronger when professional identity is weak than when it is strong [45], further highlighting the existence of an interaction between the two variables in relation to job burnout. Li’s study also suggested that professional identity can negatively predict the stress [46]. By analyzing these four models, various pension institutions can directly or indirectly relieve job burnout by improving employees’ professional identity or by reducing their work stress [47], in particular, to reduce the work stress in the consistency between revenue and responsibility areas.

### 4.3. Differences in Work Stress and Professional Identity Among Elderly Care Workers with Different Demographic Characteristics

This study demonstrated the combined effect of work stress and professional identity on burnout. We also discussed the differences in work stress and professional identity among elderly care workers with different demographic characteristics, which were also validated in prior studies [48,49,50]. The work stress score of elderly care workers was reflected in gender, number of family members, labor contract status, monthly income, compensation mechanism, income satisfaction, number of substitute pension institutions, self-assessment health, post-training, and medical insurance. Their professional identity score was associated with age, the number of family members, compensation mechanism, income satisfaction, self-assessment health, post-training, and pension insurance.

Men and workers with a monthly income of ≤2000 had higher stress regarding working conditions, reflecting differences in gender and income. This implies that male and low-income elderly care workers may have higher requirements for working conditions. This notion is also supported by Jiang’s and Leng’s research [49,51]. Some scholars considered that men may be more likely to view their work role as key to self-esteem and value work experiences for identity [52]. Another important result of our study was that professional identity in the age over 60 or under 40 was significantly higher than other age groups, which was consistent with Li [53]. Elderly care workers with family members ≤3 people had higher work stress and lower professional identity than those with >3 family members. Meanwhile, post-training workers had a higher professional identity and less work stress. This is consistent with the conclusion of the Press’s study, on medical students [54], and Noh’s findings about family members and stress levels. Those not signing labor contracts had higher work task stress than signers. However, full-time contract workers scored lower on control and work conditions [55]. This suggested that the labor contract can be both a safeguard and a restraint. Workers whose compensation was determined by the number of older people who had less stress on working tasks and working conditions and higher professional identity. Su’s study on salary categories showed that the compensation mechanism affected work stress and professional identity [56]. Workers with more substitute pension institutions had greater work stress in terms of work tasks and working conditions, which might explain their frequent job changes. Vallone’s study suggested that over 7 years of seniority was a protective factor against job change [57]. Interestingly, workers extremely satisfied or dissatisfied with income had a higher professional identity, probably because high expectations from a strong professional identity cause income dissatisfaction. Wu’s study on nurses confirmed that the difference in the professional identity of the nurses with different monthly income satisfaction was statistically significant, though the monthly income satisfaction was not an influencing factor [58].

Finally, workers with pension insurance had a higher professional identity, while those with medical insurance had less work stress. Zhao’s research on insurance and health status showed that adults with certain public insurance were more likely to report poor health [59]. Thus, we predict that pension institutions can alleviate employee job burnout indirectly by setting up an appropriate insurance system to influence work stress and professional identity.

### 4.4. Limitations

The findings in this study should be viewed in light of two key limitations. First, only elderly care workers from Heilongjiang Province were recruited, and male elderly care workers recruited in our studies were limited, limiting the generalizability of findings. The reason may be that most of the women play a role in providing care services in the family. When they lack job skills and need to find jobs, they will choose jobs such as homemaking and taking care of others [60]. Second, there was a self-designed work stress questionnaire that was not widely tested. In order to establish content validity, factor analysis and principal component analysis were used to develop an internally consistent scale and reduce items. Based on these methods, the study achieved good internal consistency (Cronbach’s α= 0.84). Moreover, the three subscale solutions accounted for 74.42% of the overall variance, which indicated that the measurement instrument displayed reasonable validity.

## 5. Conclusions

In summary, this study is an important step forward in understanding how job stress and professional identity are related to the burnout of elderly care workers. It has crucial significance for the pension institutions that elderly care workers want to use to alleviate their professional pressure or enhance their professional identity and reduce their level of job burnout. First, the study confirmed the joint effect of professional identity and job stress on burnout and the regulatory role of consistency between revenue and responsibility of work stress through four model analyzes. Pension institutions should focus more on the work stress and professional identity of elderly care workers, especially the work stress in consistency between revenue and responsibility, to alleviate job burnout. Second, the study also highlighted the significant differences in demographic economic characteristics in professional identity and the three subscales of work stress, which provided a possible basis for further research on how to reduce work stress and enhance professional identity for elderly care workers.

## 6. Implications

These research findings indicate that the job burnout, work stress, and professional identity of elderly care workers are all at a moderate level. However, it is noteworthy that in the job burnout dimension, the score for personal accomplishment is extremely low, suggesting that these workers have a poor sense of personal achievement. This aligns with Expectancy Theory: since they perceive the job to have low requirements in terms of education and other skills, their expectations from work are not particularly high. Nevertheless, this profession lacks attractiveness, which is a matter of great concern given the increasing demand for elderly care services.

Consequently, administrators should take steps to enhance the professional identity and personal accomplishment among caregivers. This can be achieved through various means. Specifically, the following strategies are recommended: First and foremost, institutions should prioritize the monitoring and management of caregivers’ work stress, especially focusing on balancing compensation with job responsibilities and ensuring fair compensation relative to job demands. Secondly, strategies such as implementing a fair compensation system aligned with caregivers’ duties, providing regular stress-management training, and fostering a supportive work environment can effectively enhance professional identity and alleviate job burnout. Future research should explore how demographic factors (e.g., age, gender, and education level) and economic indicators (e.g., income levels) influence professional identity and work stress.

## Figures and Tables

**Table 1:** The status of work stress and professional identity by basic characteristics of respondents in this study.

Characteristics		N (%)	Work Stress (t/F)			Professional Identity (t/F)
			Consistency Between Revenue and Responsibility	Work Task	Work Conditions	
Gender	Male	54 (12.3)				
	Female	385 (87.7)				
			−0.70	1.81	3.55 **	−0.13
Age	≤40	79 (18.0)				
	41–50	182 (41.5)				
	51–60	170 (38.7)				
	>60	8 (1.8)				
			1.24	2.13	1.26	2.74 *
Education level	Undergraduate and above	8 (1.8)				
	Junior college	7 (1.6)				
	High school or technical secondary school	112 (25.5)				
	Junior high school	237 (54.0)				
	Primary school and below	75 (17.1)				
			1.23	0.11	0.36	1.73
Marital status	Unmarried	10 (2.3)				
	Married	368 (83.8)				
	Divorced	37 (8.4)				
	Widowed	24 (5.5)				
			2.27	1.88	1.65	1.73
Number of family members	≤3	325 (74.0)				
	>3	114 (26.0)				
			2.80 **	2.51 *	2.32 *	−2.00 *
Labor contract signing	Have	273 (62.2)				
	No	166 (37.8)				
			−1.75	−3.11 **	−1.78	0.52
Monthly income	≤2000	213 (48.5)				
	2001–3000	179 (40.8)				
	>3000	47 (10.7)				
			2.25	0.54	4.64 *	2.30
Compensation mechanism	Length of work	20 (4.6)				
	Number of older people	255 (58.1)				
	Regular wage	164 (37.4)				
			2.91	3.64 *	11.41 **	4.29 *
Income satisfaction	Extremely satisfied	50 (11.4)				
	Basically satisfied	191 (43.5)				
	Commonly	144 (32.8)				
	Unsatisfied	51 (11.6)				
	Extremely unsatisfied	3 (0.7)				
			11.96 **	11.25 **	5.15 **	17.25 **
Number of replace pension institutions	0	318 (72.4)				
	1–2	82 (18.7)				
	≥3	39 (8.9)				
			0.46	5.11 **	4.29 *	2.22
Self-assessment health	Extremely good	56 (12.8)				
	Good	273 (62.2)				
	Commonly	95 (21.6)				
	Not good	15 (3.4)				
	Extremely not good	0 (0)				
			5.74 **	9.16 **	2.17	31.11 **
Every day in the post-time	≤10h	185 (42.1)				
	>10h	254 (57.9)				
			0.18	−0.21	1.74	−1.59
Post-training	No	87 (19.8)				
	Have	352 (80.2)				
			0.69	1.78	3.31 **	−7.71 **
Pension insurance	Have	186 (42.4)				
	No	253 (57.6)				
			−1.31	−1.70	−1.26	3.71 **
Medical insurance	Have	270 (61.5)				
	No	169 (38.5)				
			−3.85 **	−2.78 **	−2.29 *	1.69

N = 439; * *p* < 0.05; ** *p* < 0.01.

**Table 2:** Score of stress, professional identity and job burnout for elderly care workers.

Variables		The Lowest Score	The Highest Score	Average Score of Total Score (M ± SD)	Average Score of the Dimension Items (M ± SD)
Work stress		8	40	17.35 ± 5.39	
	Consistency between revenue and responsibility	4	20	9.96 ± 3.84	2.49 ± 0.96
	Work task	2	10	4.17 ± 1.44	2.08 ± 0.72
	Work conditions	2	10	3.22 ± 1.23	1.61 ± 0.62
Professional identity		10	50	35.38 ± 7.38	3.54 ± 0.74
Job burnout		0	90	24.95 ± 12.44	
	Emotional exhaustion	0	30	8.34 ± 5.06	1.67 ± 1.01
	Depersonalization	0	24	5.62 ± 3.78	1.40 ± 0.94
	Personal accomplishment	0	36	11.00 ± 6.50	1.83 ± 1.17

**Table 3:** Correlation matrix between variables.

Variables	Consistency Between Revenue and Responsibility	Work Task	Work Conditions	Professional Identity	Job Burnout
Consistency between revenue and responsibility	1	-	-	-	-
Work task	0.58 **	1	-	-	-
Work conditions	0.28 **	0.45 **	1	-	-
Professional identity	−0.37 **	−0.36 **	−0.21 **	1	-
job burnout	0.49 **	0.40 **	0.42 **	−0.48 **	1

** *p* < 0.01.

**Table 4:** Predictors of overall job burnout.

Variables	Model 1 Beta	Model 2 Beta	Model 3 Beta	Model 4 Beta
Gender	−0.06	−0.01	−0.02	−0.03
Age	0.10 *	0.09 *	0.08 *	0.08 *
Education level	−0.08	−0.05	−0.06	−0.06
Marital status	−0.02	−0.05	−0.05	−0.04
Number of family members	−0.02	0.00	0.01	−0.01
Labor contract signing	0.04	−0.01	0.03	0.02
Monthly income	−0.14 **	−0.09 *	−0.08	−0.08
Compensation mechanism	−0.09	−0.13 **	−0.13 **	−0.13 **
Income satisfaction	0.07	0.05	0.01	−0.00
Number of replace pension institutions	0.05	0.04	0.03	0.03
Self-assessment health	0.04	0.13 **	0.05	0.06
Every day in the post-time	0.07	0.04	0.06	0.05
Post-training	0.02	−0.05	0.01	0.01
Pension insurance	0.06	0.12 **	0.07	0.06
Medical insurance	0.13 **	0.04	0.07	0.06
**Professional identity**	−0.42 **		−0.30 **	−0.49 **
**Work stress**				
Consistency between revenue and responsibility		0.35 **	0.29 **	−0.29
Work task		0.00	−0.04	0.12
Work conditions		0.29 **	0.28 **	0.30
Work stress * Professional identity				
Consistency between revenue and responsibility				0.56 *
Work task				−0.14
Work conditions				−0.04
Adjusted R^2^	0.30	0.39	0.45	0.46

* *p* < 0.05; ** *p* < 0.01.

## Data Availability

The datasets generated during the current study are available from the corresponding author on reasonable request.
